# Supplementary material for: Lockdown-Associated Hunger May Be Affecting Breastfeeding: Findings from a Large SMS Survey in South Africa
Source: Int J Environ Res Public Health. 2021 Dec 30;19(1):351. doi: 10.3390/ijerph19010351 (PMC8744606; doi:10.3390/ijerph19010351)
Supplement: Supplementary file 1 [file ijerph-19-00351-s001.zip › ijerph-1407182-supplementary.pdf]

SUPPLEMENTARY MATERIAL: SURVEY QUESTIONS AND SAMPLE INFORMATION

SURVEY QUESTIONS (JUNE 2020):

**Pre-birth mothers (A)**	-	**Post-birth mothers (B)**
Hi Mama, you have been chosen for a MomConnect study to understand how the Coronavirus affects health. If you answer the questions, you will get R10 airtime.	-	Hi Mama, you have been chosen for a MomConnect study to understand how the Coronavirus affects health. If you answer the questions, you will get R10 airtime.
It will not cost you anything. To join the study reply "JOIN". To leave the study at anytime, reply "STOP". For more information, reply “MORE”		It will not cost you anything. To join the study reply "JOIN". To leave the study at anytime, reply "STOP". For more information, reply “MORE”
If they reply "JOIN" then proceed		If they reply "JOIN" then proceed
A1		B1
Thanks for joining! When did you last go to the clinic or hospital?<br><br>1-Before March<br>2-March<br>3-April<br>4-May<br>5-June<br><br>6-Don't want to answer		Thanks for joining! When did you last go to the clinic or hospital?<br><br>1-Before March<br>2-March<br>3-April<br>4-May<br>5-June<br><br>6-Don't want to answer
If respondent answers "1-3" for A1, go to A2. If not, go to A3		If respondent answers"1-3" for B1, go to B2. If not, go to B3
A2		B2
Why didn't you go to the clinic recently?<br><br>1-Waiting time<br>2-Transport problems<br>3-Afraid of getting the Coronavirus<br>4-No need<br>5-Other<br>6-Don't want to answer		Why didn't you go to the clinic recently?<br><br>1-Waiting time<br>2-Transport problems<br>3-Afraid of getting the Coronavirus<br>4-No need<br>5-Other<br>6-Don't want to answer
A3		B3
During April, May and June, was there a child in your home who was sick or needed a vaccination?<br><br>1-Yes<br>2-No<br>3–Don’t want to answer		During April, May and June, was there a child in your home who was sick or needed a vaccination?<br><br>1-Yes<br>2-No<br>3–Don’t want to answer
If respondent answers "Yes" to A3, then ask A4. If not ask A6		If respondent answers "Yes" to B3, then ask B4. If not, ask B6
A4		B4

Did the child see a nurse/doctor?<br><br>1-Yes<br>2-No<br>3-Don't want to answer	Did the child see a nurse/doctor?<br><br>1-Yes<br>2-No<br>3-Don't want to answer
If respondent answers "No" to A4, then ask A5, if not ask A6	If respondent answers "No" to B4, then ask B5. If not ask B6
A5	B5
Why didn't the child see a nurse/doctor?<br><br>1-Afraid of getting the Coronavirus<br>2-No need<br>3-Waiting time<br>4-Transport problems<br>5-Other<br>6-Don't want to answer	Why didn't the child see a nurse/doctor?<br><br>1-Afraid of getting the Coronavirus<br>2-No need<br>3-Waiting time<br>4-Transport problems<br>5-Other<br>6-Don't want to answer
A6	B6
If you take ART, during May and June, have you run out of medication?<br><br>1-Yes<br>2-No<br>3-Don't need ART<br>4-Don't want to answer	If you take ART, during May and June, have you run out of medication?<br><br>1-Yes<br>2-No<br>3-Don't need ART<br>4-Don't want to answer
If respondent answers "Yes" to A6, then ask A7, if not. If not ask A8	If the respondent answers "Yes" to B6, then ask B7. If not ask B8
A7	B7
Why did you run out of ART?<br><br>1-Afraid of getting the Coronavirus<br>2-No ART available<br>3-Waiting time<br>4-Transport problems<br>5-Don't want to answer	Why did you run out of ART?<br><br>1-Afraid of getting the Coronavirus<br>2-No ART available<br>3-Waiting time<br>4-Transport problems<br>5-Don't want to answer
A8	B8
Has any adult in this household gone to bed hungry in the past 7 days?<br><br>1-Yes<br>2-No<br>3-Don't want to answer	Has any adult in this household gone to bed hungry in the past 7 days?<br><br>1-Yes<br>2-No<br>3-Don't want to answer
A9	B9

Has any child in this household gone to bed hungry in the past 7 days?<br><br>1-Yes<br>2-No<br>3- No child in the household<br>4-Don't want to answer		Has any child in this household gone to bed hungry in the past 7 days?<br><br>1-Yes<br>2-No<br>3- No child in the household<br>4-Don't want to answer
		B10
		Have you applied for a Child Support Grant for your new baby?<br><br>1-Yes<br>2-No<br>3-Don't want to answer
		If yes to B10, then B11. If not, then ask B12
		B11
		Have you started receiving any Child Support Grant payment yet for your new baby?<br><br>1-Yes<br>2-No<br>3-Don't want to answer
A10		B12
Is anyone in your household receiving a Child Support Grant or an Old Age Pension?<br><br>1-Yes<br>2-No<br>3-Don't want to answer		Is anyone in your household receiving a Child Support Grant or an Old Age Pension?<br><br>1-Yes<br>2-No<br>3-Don't want to answer
A11		B13
In the last 7 days have you felt hopeless, down or depressed?<br>1-No<br>2-Yes, for a few days<br>3-Yes, for most days<br>4-Don't want to answer		In the last 7 days have you felt hopeless, down or depressed?<br>1-No<br>2-Yes, for a few days<br>3-Yes, for most days<br>4-Don't want to answer
A12		B14
In the last 7 days have you felt little interest or pleasure in doing things?<br>1-No<br>2-Yes, a few days<br>3-Yes, most days<br>4-Don't want to answer		In the last 7 days have you felt little interest or pleasure in doing things?<br>1-No<br>2-Yes, a few days<br>3-Yes, most days<br>4-Don't want to answer
END		END

Thank you! If you need help in these difficult times, contact Lifeline 0861 322 322 or National Mental Health Information Line at 0800 567 567.	Thank you! If you need help in these difficult times, contact Lifeline 0861 322 322 or National Mental Health Information Line at 0800 567 567.

SURVEY QUESTIONS (JULY 2020):

**MATCH Wave 2: to be adminsitered to the SAME mothers as Wave 1**	
**Pre-birth mothers (A)**	**Post-birth mothers (B)**
Hi Mama, thanks for completing the last survey on MomConnect, we'd like to ask you a few more questions. If you answer the questions, you will get another R10 airtime.	Hi Mama, thanks for completing the last survey on MomConnect, we'd like to ask you a few more questions. If you answer the questions, you will get another R10 airtime.
It will not cost you anything. To join the study reply "JOIN". To leave the study at anytime, reply "STOP". For more information, reply "MORE"	It will not cost you anything. To join the study reply "JOIN". To leave the study at anytime, reply "STOP". For more information, reply "MORE"
If they reply "JOIN" then proceed	If they reply "JOIN" then proceed
**A1**	**B1**
Did you use a pregnancy test to see if you were pregnant?<br><br>1-Yes<br>2- No<br><br>9- I don't want to answer	Yesterday did you breastfeed your baby?<br>1- Yes<br>2- No<br>9- Don't want to answer
**If <u>yes</u> then ask...A2**	**B2**
Where did you use the pregnancy test?<br>1- At home<br>2- At the clinic or hospital<br>3- Somewhere else<br>9 I don't want to answer	Yesterday did you feed your baby formula or porridge such as nestum?<br>1- Yes<br>2- No<br>9- Don't want to answer
**If <u>no</u> then ask A3**	**If Yes to B1 and No to B2 then ask B3:**
Why did you not use a pregnancy test?<br><br>1- I already knew I was pregnant<br>2- I couldn't afford one<br>3- It's difficult to get one<br>4- Other<br>8-Don't want to answer	In the last 7 days did you feed your baby...<br>1- ONLY breastmilk<br>2- ONLY baby formula/nestum<br>3- Breastmilk AND baby formula/nestum<br>8-Don't want to answer
**A4**	**B4**

Do you have any other children?<br>0- No, this is my first<br>1- Yes, 1 child<br>2- Yes, 2 children<br>3- Yes, 3 children<br>4- Yes, 4 or more children<br>8- Don't want to answer	Do you have any other children?<br>0- No only my baby<br>1- Yes, 1 child<br>2- Yes, 2 children<br>3- Yes, 3 children<br>4- Yes, 4 or more children<br>8- Don't want to answer
**A5**	**B5**
In the last 7 nights did YOU ever go to bed hungry?<br>1- Yes<br>2- No<br>3- Don't know<br>9- Don't want to answer	In the last 7 nights did YOU ever go to bed hungry?<br>1- Yes<br>2- No<br>3- Don't know<br>9- Don't want to answer
**If Yes to A5 then ask A6**	**If Yes to B5 then ask B6**
**A6**	**B6**
How many nights did YOU go to bed hungry?<br>0- Never<br>1- 1<br>2- 2<br>3- 3<br>4- 4<br>5- 5<br>6- 6<br>7- 7<br>8- Don't know<br>9- Don't want to answer	How many nights did YOU go to bed hungry?<br>0- Never<br>1- 1<br>2- 2<br>3- 3<br>4- 4<br>5- 5<br>6- 6<br>7- 7<br>8- Don't know<br>9- Don't want to answer
**A7**	**B7**
In the last 7 nights did A CHILD go to bed hungry in your household?<br>1- Yes<br>2- No<br>3- Don't know<br>8- No children<br>9- Don't want to answer	In the last 7 nights did A CHILD go to bed hungry in your household?<br>1- Yes<br>2- No<br>3- Don't know<br>8- No children<br>9- Don't want to answer
**If YES to A7 the ask A8**	**If YES to B7 the ask B8**
**A8**	**B8**

How many nights did the child go to bed hungry?<br>0- Never<br>1- 1<br>2- 2<br>3- 3<br>4- 4<br>5- 5<br>6- 6<br>7- 7<br>8- Don't know<br>9- Don't want to answer	How many nights did the child go to bed hungry?<br>0- Never<br>1- 1<br>2- 2<br>3- 3<br>4- 4<br>5- 5<br>6- 6<br>7- 7<br>8- Don't know<br>9- Don't want to answer
**A9**	**B9**
Have you eaten any meat in the last 30 days?<br>1-Yes<br>2-No I can't afford meat<br>3-No, I didn't want meat<br>5-Don't Know,<br>6- Don't want to answer	Have you eaten any meat in the last 30 days?<br>1-Yes<br>2-No I can't afford meat<br>3-No, I didn't want meat<br>5-Don't Know,<br>6- Don't want to answer
**A10**	**B10**
Since you found out you were pregnant how many times have you visited a clinic?<br>0- Never<br>1- 1 time<br>2- 2 times<br>3- 3 times<br>4- 4 times<br>5- 5 times<br>6- 6 or more<br>8- Don't know<br>9- Don't want to answer	Since your baby was born, have you ever taken your baby to the clinic?<br>1-Yes<br>2-No<br><br>8- Don't know<br>9- Don't want to answer
**A11**	**If YES to B10 then ask B11**
Do you think you are likely to get the coronavirus?<br><br>1- Yes<br>2-No<br><br>8-Don't know<br>9-Don't want to answer	How many times have you taken your baby to the clinic since they were born?<br>0- 0<br>1- 1<br>2- 2<br>3- 3<br>4- 4<br>5- 5<br>6- 6 or more times<br>8- Don't know<br>9- Don't want to answer

A12	B12
In June did you have any kind of job or sell things or earn any income, no matter how small?<br>1- Yes<br>2- No<br>9-Don't want to answer	When did your child last receive a vaccination/umjovo?<br><br>1-Before March<br>2-March<br>3-April<br>4-May<br>5-June 6-July<br>8-Don't know<br>9-Don't want to answer
**A13**	**B13**
What is your highest level of education?<br>1- Completed primary school<br>2- Some high school<br>3- I've passed matric<br>4- Tertiary qualification<br>9-Don't want to answer	What is your highest level of education?<br>1- Completed primary school<br>2- Some high school<br>3- I've passed matric<br>4- Tertiary qualification<br>9-Don't want to answer
**A14 Open-ended question**	**B14 Open-ended question**
This is the last question, what is the one thing you are MOST worried about at the moment? Please type your answer...	This is the last question, what is the one thing you are MOST worried about at the moment? Please type your answer...
END	END
Thank you! If you need help in these difficult times, contact Lifeline 0861 322 322 or National Mental Health Information Line at 0800 567 567.	Thank you! If you need help in these difficult times, contact Lifeline 0861 322 322 or National Mental Health Information Line at 0800 567 567.

SAMPLE INFORMATION:

	**mean**
**Province**	
Eastern Cape	7%
Free State	5%
Gauteng	26%
KwaZulu-Natal	19%
Limpopo	16%
Mpumulanga	12%
NorthWest	7%
Northern Cape	1%

Western Cape	6%
**Post-birth**	43%
**Socioeconomic status**	
Quntile 1	21%
Quntile 2	21%
Quntile 3	17%
Quntile 4	18%
Quntile 5	23%
**Age of mother**	
18 and 19	5%
20-29	64%
30-39	29%
40-49	2%

For 2287 women who respponded tothe first and second survey
